# Approaching unsafe limits: climate-related health inequities within and beyond Europe

**DOI:** 10.1016/j.lanepe.2023.100683

**Published:** 2023-07-13

**Authors:** Kim R. van Daalen, Cathryn Tonne, Carme Borrell, Maria Nilsson, Rachel Lowe

**Affiliations:** a Barcelona Supercomputing Center (BSC), Barcelona, Spain; b Barcelona Institute for Global Health (ISGlobal), Barcelona, Spain; c Universitat Pompeu Fabra (UPF), Barcelona, Spain; d CIBER Epidemiología y Salud Pública (CIBERESP), Madrid, Spain; e Agència de Salut Pùblica de Barcelona (ASPB), Barcelona, Spain; f Sant Pau Biomedical Research Institute (IIB Sant Pau), Barcelona, Spain; g Department of Epidemiology and Global Health, Umeå University, Sweden; h Centre on Climate Change and Planetary Health, London School of Hygiene and Tropical Medicine, London, UK; i Centre for Mathematical Modelling of Infectious Diseases, London School of Hygiene and Tropical Medicine, London, UK; j Catalan Institution for Research and Advanced Studies (ICREA), Barcelona, Spain

For the first time in history, it is estimated that it is more likely than not that global near-surface temperatures will temporarily overshoot 1.5 °C above pre-industrial levels before 2028; demonstrating that the world is edging closer to the 1.5 °C planetary boundary and is failing to adequately cut emissions.^1^ Simultaneously, sea temperatures are rising remarkably (2023), with expected further increases due to emerging El Niño conditions (the warm phase of the El Niño-Southern Oscillation) which will likely aggregate extreme weather worldwide. In Europe, temperatures are soaring twice the global average, with past decades characterised by unprecedented warming (Fig. 1) and escalating extreme conditions. Countries are now bracing themselves for another ferocious summer dominated by heat stress, droughts, water shortages, and farmers losing their harvests.

**Fig. 1:**
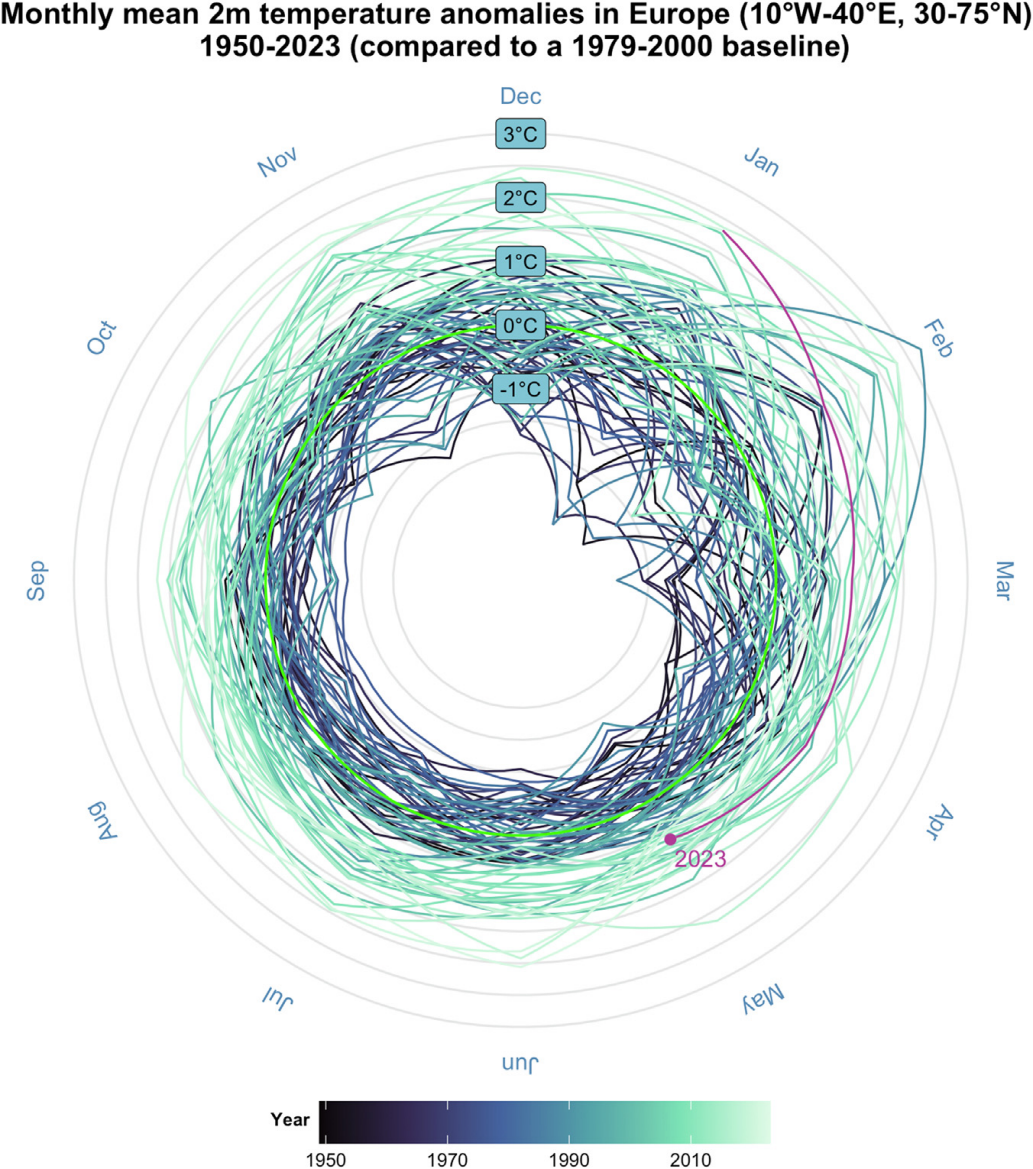
**Monthly mean temperature anomalies in Europe between 1950 and 2023.** Anomalies are defined relative to a base period of 1979–2000. Lines represent the monthly mean 2 m temperature anomalies in Europe between January 1950 and May 2023, with more recent years represented by a lighter blue and years further in the past represented by a darker blue (see legend). The bright green line represents 0 C° of change relative to the base period. The purple line represents 2023 up to May. These temperatures are based on 2 m temperature data from ECMWF ERA5, and are the temperature of the air at 2 m above the surface of land, sea or inland waters. 2 m temperature is calculated by interpolating between the lowest model level and the Earth’s surface, taking account of the atmospheric conditions. The bounding box for Europe was defined at 10°W–40°E, 30–75°N. The figure was made using R (version 4.0.5) ggplot and dplyr packages using publicly available code (https://earth.bsc.es/gitlab/ghr/temperature-spiral).

Climate change is not a far-in-the-future theoretical scenario; it is here, it kills, and it is worsening. For years, scientists have emphasised the increasing frequency and scale of climate-related health impacts,^2^ and the harmful air pollution caused by many climate change drivers.^2^^,^^3^ From heat-related morbidity to altered environmental suitability for infectious diseases; negative health trends are expected to accelerate and intensify under all emission scenarios.^3^^,^^4^ Not unexpectedly, health risks are projected to be less severe at lower compared to higher levels of global warming.^3^, ^4^, ^5^ Furthermore, the next 0.5 °C global increase above “today” (1 °C, 2017) is suggested to involve greater risk per unit temperature than the last 0.5 °C increase: risks are accelerating.^5^ Events similar to the European 2003 summer—characterised by thousands of deaths and severe socio-economic and environmental impacts—are estimated to be at least 24% more frequent at 2 °C compared to 1.5 °C warming.^6^ Rising temperatures are also projected to shift and expand areas climatically suitable for infectious disease transmission in Europe.^3^ Limiting warming to 1.5 °C averts some of the worst health impacts.^3^, ^4^, ^5^

But climate-related health impacts are not equal, nor is responsibility for climate change. Populations within and between regions are differentially impacted due to, among others, differences in land and ocean use, income and wealth inequality, socio-economic development, social hierarchies and marginalisation, and historical and ongoing legacies of colonialism.^4^^,^^7^ Populations most impacted tend to be least responsible, less likely to be recognised or prioritised, and burdened with addressing multiple priorities simultaneously.^7^ Whilst Global South populations are most severely impacted; disproportionate negative health impacts also fall upon minoritised and disadvantaged European communities.

Countries, corporations and the ruling class within the EU—containing some of the wealthiest countries globally that benefited most from industrialization, capitalism and colonialism—are major contributors to historical and current GHG emissions,^2^^,^^7^ whilst outsourcing many environmental pressures and negative impacts related to EU consumption to places outside the EU.^8^ Illustratively, EU countries tend to import goods and services from China, Eastern European neighbours, and other countries. This results in GHG emissions, water consumption, ecotoxicity, local air pollution, related premature mortality, and other local adverse health impacts.^8^^,^^9^ Climate change is inherently a social and health justice problem.^7^

Within Europe, differential exposure, sensitivity, and adaptive capacity results in uneven distributions of climate-related health impacts—often reflecting socio-demographic inequities, and marginalisation. On a sub-regional scale, Eastern Europe tends to experience more air pollution, whilst Southern Europe experiences more heat and climate-sensitive infectious disease suitability.^2^^,^^10^ Within countries, the health of low-income communities, migrants and displaced people, ethnic minoritised and Indigenous peoples, elderly, children, and those with health conditions are more adversely impacted, as well as the health of sexual and gender minoritised people and women going through pregnancy and childbirth. Outdoor workers, often of disadvantaged socio-economic position or migrants, are more likely to be affected by climate-related health risks due to increased exposure to climate hazards. Yet, thus far, environmental equity, including addressing disproportionate socio-spatial distributions of environmental exposures and health risks, is not an explicit goal within EU policies.^10^

Eight years after committing to limit global temperature increase to below 2 °C, mitigation policies are still either inadequate or lacking, highlighted by a projected 3.2 °C increase by 2100.^4^ Some progress was made with the adoption of the European Climate Law: the legal basis for net-zero by 2050.^2^ However, based on the latest IPCC report,^11^ the EU should aim to reach net-zero earlier: as close as possible to 2040. More so, when using Finland’s approach, based on the country’s fair share of emissions (calculated using Finland’s population and historical emissions) instead of how fast economies can transition, the EU should reach net-zero early to mid-2030s to limit warming to 1.5 °C more fairly.^12^ Importantly, more ambitious mitigation can deliver large global health gains aside from averting further climate change, with particularly evident health co-benefits for air quality. The EU Ambient Air Quality Directive revision is a crucial opportunity to ensure climate mitigation and air quality goals align. By going beyond currently proposed limits and committing to reduce air pollution in line with the WHO health-based Air Quality Guidelines, the EU can provide strong impetus to phase out fossil fuels, whose use is incompatible with safe air.^13^ Furthermore, albeit a step forward with the Carbon Border Adjustment Mechanism adoption, European climate and air pollution targets tend to focus solely on domestic emissions and pollution leaving tremendous loopholes for outsourcing negative environmental and health impacts elsewhere.

We can’t keep treating climate change symptoms without addressing the underlying inequities between and within countries. Europe should commit to guaranteeing a fair and healthy environmental transition including further slashing Europe’s emissions, taking global responsibility, and supporting those mostly affected within and beyond Europe.

## Contributors

KRvD and RL conceived the presented idea and wrote the first draft of the manuscript, and all other authors contributed to subsequent versions. KRvD generated the figure. All authors reviewed and approved the final version of the manuscript.

## Declaration of interests

The authors have no conflict of interest to declare.

## References

[1] World Meteorological Organization (2023). https://library.wmo.int/doc_num.php?explnum_id=11629.

[2] van Daalen K.R., Romanello M., Rocklöv J. (2022). The 2022 Europe report of the Lancet countdown on health and climate change: towards a climate resilient future. Lancet Public Health.

[3] Ebi K.L., Hasegawa T., Hayes K., Monaghan A., Paz S., Berry P. (2018). Health risks of warming of 1.5 °C, 2 °C, and higher, above pre-industrial temperatures. Environ Res Lett.

[4] IPCC (2022).

[5] Hoegh-Guldberg O., Jacob D., Taylor M. (2019). The human imperative of stabilizing global climate change at 1.5 °C. Science.

[6] King A.D., Karoly D.J. (2017). Climate extremes in Europe at 1.5 and 2 degrees of global warming. Environ Res Lett.

[7] Deivanayagam T.A., English S., Hickel J. (2023). Envisioning environmental equity: climate change, health, and racial justice. Lancet.

[8] Bruckner B., Shan Y., Prell C. (2023). Ecologically unequal exchanges driven by EU consumption. Nat Sustain.

[9] Nansai K., Tohno S., Chatani S. (2021). Consumption in the G20 nations causes particulate air pollution resulting in two million premature deaths annually. Nat Commun.

[10] Ganzleben C., Kazmierczak A. (2020). Leaving no one behind - understanding environmental inequality in Europe. Environ Health.

[11] Core Writing Team, Lee H., Romero J. (2023). Climate Change 2023: Synthesis Report. A Report of the Intergovernmental Panel on Climate Change. Contribution of Working Groups I, II and III to the Sixth Assessment Report of the Intergovernmental Panel on Climate Change.

[12] Ollikainen M., Weaver S., Seppälä J. (2019).

[13] Boogaard H., Andersen Z.J., Brunekreef B. (2023). Clean air in Europe for all: a call for more ambitious action. Environ Epidemiol.

